# Genetic polymorphisms in PXR and NF-κB1 influence susceptibility to anti-tuberculosis drug-induced liver injury

**DOI:** 10.1371/journal.pone.0222033

**Published:** 2019-09-06

**Authors:** Jingwei Zhang, Zhenzhen Zhao, Hao Bai, Minjin Wang, Lin Jiao, Wu Peng, Tao Wu, Tangyuheng Liu, Hao Chen, Xingbo Song, Lijuan Wu, Xuejiao Hu, Qian Wu, Juan Zhou, Jiajia Song, Mengyuan Lyv, Binwu Ying

**Affiliations:** 1 Department of Laboratory Medicine, West China Hospital, Sichuan University, Chengdu, China; 2 West China School of Medicine, Sichuan University, Chengdu, China; National Institutes of Health, UNITED STATES

## Abstract

**Background:**

Pregnane X receptor (PXR) regulates the expression of drug-metabolizing enzymes and transport enzymes. NF-κB not only plays a role in liver homeostasis and injury-healing processes by regulating inflammatory responses but may also regulate the transcription of PXR. Currently, genetic polymorphisms in PXR are associated with adverse drug effects. Because little is known about the association between NF-κB1 genetic polymorphisms and adverse drug reactions, we explored the association between PXR and NF-κB1 single nucleotide polymorphisms (SNPs) and susceptibility to anti-tuberculosis drug-induced liver injury (ATDILI).

**Materials and methods:**

A total of 746 tuberculosis patients (118 with ATDILI and 628 without ATDILI) were prospectively enrolled at West China Hospital between December 2014 and April 2018. Nine selected SNPs (rs3814055, rs13059232, rs7643645 and rs3732360 in PXR and rs78872571, rs4647992, rs60371688, rs1598861 and rs3774959 in NF-κB1) were genotyped with a custom-designed 2x48-plex SNP Scan TM Kit. The frequencies of the alleles, genotypes and genetic models of the variants were compared between patients with or without ATDILI, while joint effect analysis of the SNP-SNP interactions was performed using multiplicative and additive models. The odds ratios (ORs) and the corresponding 95% confidence intervals (CIs) were calculated.

**Results:**

The T allele of rs3814055 in PXR was associated with a decreased risk for ATDILI (OR 0.61; 95% CI: 0.42–0.89, p = 0.0098). The T alleles of rs78872571 and rs4647992 in NF-κB1 were significantly associated with an increased risk for ATDILI (OR 1.91; 95% CI: 1.06–3.43, p = 0.028 and OR 1.81; 1.06–3.10, p = 0.029, respectively). The allele, genotype and genetic model frequencies were similar in the two groups for the other six SNPs (all P>0.05). There were no multiplicative or additive interactions between the SNPs.

**Conclusion:**

Our study is the first to reveal that rs3814055 variants in PXR and rs78872571 and rs4647992 variants in NF-κB1 are associated with susceptibility to ATDILI caused by first-line anti-tuberculosis combination treatment in the Han Chinese population.

## Introduction

Tuberculosis remains a serious hazard to human health. In 2017, approximately 1.7 billion people were infected with *Mycobacterium* *tuberculosis*, and approximately 1.6 million deaths were caused by tuberculosis worldwide. The number of tuberculosis infections in China accounts for approximately 9% of the total number worldwide[1]. Early diagnosis, effective treatment and the prevention of the development of drug-resistant bacteria are key to block the spread of tuberculosis. At present, WHO still recommends the combination of isoniazid, rifampicin, pyrazinamide and streptomycin as the first-line treatment protocol [1]. Although effective, the side effects of the combination protocol cannot be ignored, and anti-tuberculosis drug-induced liver injury (ATDILI) is the most common side effect, with an incidence of 2.0–28.0%[2]. The early symptoms of ATDILI are atypical and the diagnostic criteria lack specificity, which can cause a missed diagnosis or misdiagnosis, delaying treatment and resulting in adverse consequences. Severe ATDILI can even cause fulminant hepatic failure or death, resulting in a heavy social burden[3]. It is necessary and urgent to clarify the pathogenesis of ATDILI and identify the key molecules involved in its progression as targets for diagnosis and treatment.

The pathogenesis of ATDILI mainly involves drug metabolism, oxidative stress, mitochondrial dysfunction, immune dysregulation and inflammatory responses[4, 5]. However, there are still many unknown factors involved in the complex and sophisticated regulatory network that underlies the development of ATDILI. As ATDILI is idiosyncratic, there is growing evidence that the genetic mutation of related genes may be involved in its pathogenesis. Single nucleotide polymorphisms (SNPs), which serve as third-generation genetic markers, have been shown to have value for the guidance of the clinical treatment of ATDILI. The association of the “slow acetylation” phenotype, which involves the NAT gene, with an increased rate of toxic reactions is now included in the FDA drug label for isoniazid, which is used for the treatment of tuberculosis[6]. Other genes, such as cytochrome enzyme P450 2E1 (CYP2E1), cytochrome enzyme P450 7A1 (CYP7A1), bile salt export pump (BSEP), UDP glucuronosyl transferase A1(UGT1A1), pregnane X receptor (PXR), human leukocyte antigens (HLAs), tumour necrosis factor-α (TNF-α) and superoxide dismutases (SODs), have also been widely investigated to clarify their potential roles in ATDILI[6]. Because these studies were usually limited to a certain population or sample size, their conclusions usually lacked universality and required further verification to some degree; however, these studies also provided new insights and presented pioneering work to spur further research.

Pregnane X receptor (PXR), also known as nuclear receptor 1I2 (NR1I2), is a member of the nuclear receptor superfamily of ligand-dependent transcription factors. The primary function of PXR is to regulate the expression of drug-metabolizing and transport enzymes to prevent the accumulation of toxic chemicals in the liver to maintain homeostasis[7]. In addition, PXR can act as a regulatory gene for cholesterol and bile acid metabolism and the expression of heme- and inflammatory response-related genes[8, 9]. The dysregulation of PXR and downstream genes may lead to decreased therapeutic efficacy and/or increased toxicity in the liver [10]. As rifampicin is a typical ligand of PXR, ligand-receptor interactions between rifampicin and PXR could trigger the expression of various genes, including those involved in xenobiotic oxidation, conjugation and transportation[5] [11]. In vivo, high concentrations of hepatoxic protoporphyrin IX were noted in the livers of PXR–humanized (hPXR) mice, which provided clues regarding PXR-mediated liver injury caused by rifampicin and isoniazid co-therapy [12]. Genetic polymorphisms of the PXR gene have been speculated to play an important role in adverse drug effects, such as flucloxacillin-induced liver injury [9] [13]. Overall, genetic polymorphisms of PXR may be associated with the risk of ATDILI resulting from rifampicin and isoniazid anti-tuberculosis co-therapy. Some PXR variants have been shown to increase susceptibility to ATDILI in an Indonesian population and a Chinese population, although the results were inconsistent and require further verification[14–16].

Nuclear factor-kappaB **(**NF-κB**)** is a member of the Rel family, which includes RelAp (65), RelB, cRel, NF-κB1 (p50 and p105) and NF-κB2 (p52 and p100) [17]. NF-κB plays an essential role in the regulation of inflammatory signalling pathways and contributes to liver homeostasis and injury-healing processes [18]. NF-κB is activated in many liver diseases, including alcoholic liver disease, nonalcoholic fatty liver disease, viral hepatitis and biliary liver disease. A study has shown that tumour necrosis factor-α (TNF-α) stimulates hepatocytes to release reactive oxygen species (ROS), which in turn leads to hepatocyte injury by activating NF-κB signalling [19]. In addition to regulating inflammatory reactions, as a transcription factor, NF-κB can regulate the transcription of genes involved in drug-induced liver injury, such as UDP glucuronosyl transferase A1 (UGT1A1) and PXR. NF-κB can directly bind to the upstream promoter region (-725/-716) of the human UGT1A1 gene, which may partly explain the molecular pathogenesis of inflammation-associated hyperbilirubinaemia[20]. In addition, the activation of NF-κB can suppress the activation of various NRs, including PXR [21]. Although it is involved in multiple pathways and genes related to ATDILI, the mechanism that causes an NF-κB signalling path imbalance to lead to ATDILI remains unclear. When it is inactive, NF-κB binds to its inhibitory protein in the cytoplasm. After stimulation, the inhibitory protein is degraded, and then the activated heterodimer is released and transported into the nucleus, thereby initiating the expression of the target gene. The p50/p65 heterodimer is the most common complex with the highest content and is present in almost all cells in the body[22]. The NF-κB1 gene located at 4q24 encodes both the p105 and p50 subunits by alternative splicing[23]. Therefore, the NF-κB1 gene is one of the key genes in the NF-κB signalling pathway. There have been many studies on the association of genetic polymorphisms of the NF-κB1 gene with immune-related diseases such as inflammatory bowel disease[24], infectious diseases[25] and tumours[17, 23, 26]. There is evidence that NF-κB1 may directly interact with the DNA-binding domain of PXR to suppress its expression and inhibit its transactivation[27]. However, no research has been reported on the association of NF-κB1 genetic polymorphisms with liver damage caused by anti-tuberculosis drugs.

Because of the heavy burden caused by tuberculosis in China, the aim of the present study was to explore the possible association between genetic polymorphisms in PXR and NF-κB1 and the risk of ATDILI by conducting a prospective case-to-control study in the Han Chinese population.

## Subjects and methods

### Subjects

Ethical approval for this study was obtained from the Institutional Review Board of the West China Hospital of Sichuan University. We consecutively recruited 1060 patients who were highly suspected of having TB at West China Hospital between December 2014 and April 2018. In total, the presence of TB in 817 patients was confirmed by experienced respiratory physicians, who provided a clear tuberculosis diagnosis. All patients underwent standard short-course chemotherapy consisting of isoniazid, rifampicin, pyrazinamide and ethambutol for six months in accordance with the approved guidelines. The treatments were adjusted accordingly if any patient developed definitive ATDILI.

The inclusion criteria for ATDILI group were as follows: (a) normal serum alanine aminotransferase (ALT) (0–40 IU/L) and aspartate aminotransferase (AST) (0–40 IU/L) values before treatment; (b) ALT and/or AST levels ≥ 3 × upper limit of normal (ULN) (120 IU/L) with hepatitis symptoms such as jaundice, nausea, vomiting, and abdominal pain; (c) ALT and/or AST levels ≥ 5 × ULN (200 IU/L) with or without symptoms; (d) total bilirubin (TBIL) ≥ 1.5 × ULN (42 μmol/L); (e) no administration of other potentially hepatotoxic drugs two weeks before the occurrence of ATDILI; (f) no history of infection with hepatitis B virus (HBV), hepatitis C virus (HCV) or human immunodeficiency virus (HIV) before treatment[28, 29]. The inclusion criteria for the non-ATDILI group were normal serum ALT, AST and TBIL values before and after treatment.

Ultimately, 746 tuberculosis patients were enrolled in our study. The process of enrolment is shown in S1 Fig. The demographic and clinical characteristics of the enrolled patients were obtained from electronic medical records. Blood samples were collected for genotyping. Along with treatment, biochemical and haematological analyses were performed twice a month during the first two months and monthly in the subsequent four months. Test results and clinical symptoms were recorded to assess ATDILI.

### The clinical definition and severity of ATDILI

The definition of drug-induced liver injury was made according to the National Institutes of Health and Common Toxicity Criteria for Adverse Events v5.0 (CTCAE v5.0) unless stated otherwise[28]. The severity of hepatotoxicity was classified into three major categories according to the WHO Toxicity Classification Standards: Grade 1 (mild), ALT< ×5 ULN(200 IU/L); Grade 2 (moderate), ALT level higher than 5 ULN but less than 10 ULN; Grade 3 (severe), ALT level ≥ 10 × ULN (400 IU/L)[30].

### Candidate single nucleotide polymorphism selection and genotyping

The candidate SNPs in the PXR and NF-κB1 genes were selected according to the following procedure. Searches of the dbSNP (http://www.ncbi.nlm.nih.gov/projects/SNP/) and 1000 Genomes (http://www.1000genomes.org/) databases were used to find SNPs with minor allele frequencies ≥ 0.02 and a linkage disequilibrium (LD) -r^2^≥0.8 among Han Chinese from Beijing at locations within 2 kb upstream and 1 kb downstream of the start site in the genomic region [31]. SNPs are under the experimental conditions for genotyping.The experimental conditions for genotyping were determined. Functional SNPs were identified in exons, promoters, or untranslated regions (UTRs) preferentially, followed by the identification of tag SNPs that could represent three SNPs in the intron region. This implied that the SNPs altered the expression or activity of PXR, NF-κB1 or its targets in previous studies. Nine SNPs were eventually included and genotyped. Detailed information on the genotyped SNPs, including the chromosomal locations and minor allele frequencies, is summarized in S1 Table. Genomic DNA was extracted from three millilitres of EDTA-anticoagulated whole blood obtained from each participant with a QIAamp^®^ DNA blood mini kit (Qiagen, Germany) according to the manufacturer's instructions. The SNP genotyping work was conducted with a custom-designed 2x48-plex SNP scan TM Kit (Cat#:G0104, Genesky Biotechnologies Inc., Shanghai, China), which is based on patented SNP genotyping technology that utilizes double ligation and multiplex fluorescence polymerase chain reaction, as described previously[32]. To ensure the repeatability and stability of the genotyping, 30 samples were randomly selected for double-blind experiments, and all the genotype calling success rates were greater than 99.0%.

### Statistical analysis

The demographic and clinical data of the enrolled patients in the ATDILI group and the non-ATDILI group were compared using the Chi-square test and t-test by SPSS software version 17.0. The comparison of the composition ratios between the different groups was determined by a nonparametric test with Kruskal-Wallis one-way ANOVA by SPSS software version 17.0. The Hardy-Weinberg equilibrium (HWE) for all control SNPs was assessed by Plink software version 1.07. The associations between the SNPs and ATDILI were evaluated using unconditional logistic regression after adjusting for age and gender by PLINK software version 1.07. The odds ratio (OR) with the corresponding 95% confidence interval (CI) was used as a measure of association. The linkage disequilibrium (LD) and haplotype analysis were conducted with Haplotype software version 4.2, and the logistic regression model in SPSS was used to evaluate the interactions in the multiplicative model. The significance test of the regression model was estimated according to the Bootstrap method. The additive interactions between two points were evaluated using an Excel file created by Andersson et al[33].

## Results

### Demographic data and clinical characteristics of the subjects

A total of 746 TB patients were included in this study. The overall incidence rate of ATDILI among the patients was 15.82% (118/746). No differences in age (p = 0.285) and gender (p = 0.801) were found between the two groups. All patients who developed ATDILI during treatment (ATDILI group) presented alterations in hepatic enzymes, and 32 individuals developed symptomatic hepatitis, which was characterized by jaundice, nausea, vomiting and abdominal pain. Compared with the non-ATDILI group (patients without ATDILI resulting from treatment), the ATDILI group showed a higher likelihood of fever and weight loss (p = 0.016 and p = 0.036) and a higher risk of intrapulmonary tuberculosis with extra-pulmonary tuberculosis (24/118, 20.34% versus 65/628, 10.35%, p<0.001). The ATDILI group also had a higher risk of elevated serum levels of ALT (p<0.001), AST (p<0.001), ALP (p<0.001), TBIL (p = 0.002), IBIL (P = 0.049), uric acid (p = 0.008) and GGT (p = 0.021). The demographic, clinical characteristics and laboratory indicators of the patients are depicted in Table 1.

**Table 1:** Demographic, clinical characteristics and laboratory indicators of the enrolled patients.

Group	Non-ATDILI (n = 628)		ATDILI (n = 118)				P value	
**General data**								
Age (years)	40.92±15.72		42.85±18.44				0.284	
Gender (male/female)	375(59.71%)	253(40.28%)	69(58.47%)		49(41.52%)		0.801	
Smoking (No/Yes)	407(64.80%)	221(35.19%)	80(67.79%)		38(32.20%)		0.532	
Drinking (No/Yes)	465(74.04%)	163(25.95%)	83(70.33%)		35(29.66%)		0.464	
TB subtype, n (%)								
PTB	520	82.80%	79		66.95%		**<0.001**	
EPTB	43	6.85%	15		12.71%			
PTB & EPTB	65	10.35%	24		20.34%			
General symptoms (No/Yes)	135(19.62%)	492(80.37%)	23(19.49%)		95(80.51%)		0.567	
Fever (No/Yes)	344(54.78%)	284(45.22%)	50(42.37%)		68(57.62%)		***0*.*016***	
Weight loss (No/Yes)	367(58.43%)	261(41.56%)	82(69.49%)		36(30.50%)		***0*.*036***	
Night sweat (No/Yes)	433(68.94%)	195(31.05%)	86(72.88%)		32(27.12%)		0.446	
Fatigue (No/Yes)	462(73.57%)	166(26.43%)	85(72.03%)		33(27.97%)		0.716	
Poor appetite (No/Yes)	374(59.55%)	254(40.45%)	69(58.47%)		49(41.52%)		0.859	
Local infection (No/Yes)	134(21.34%)	494(78.66%)	24(20.34)		94(79.66%)		0.758	
**Laboratory values**	mean ±SD or P50 (P25-P75)							
RBC (×10^12^/L)	4.28 ± 0.68		4.31 ± 0.74				0.481	
HB (g/L)	122.06 ± 20.58		122.87 ± 22.11				0.717	
HCT (L/L)	0.36 ± 0.06		0.38 ± 0.06				0.069	
PLT (×10^9^/L)	232.50(172.75–297.25)		236.50(184.00–321.75)				0.134	
WBC (×10^9^/L)	6.51(5.17–8.44)		6.57(4.99–7.96)				0.761	
Neutrophil (×10^9^/L)	5.10 ± 2.73		5.23 ± 2.89				0.631	
Monocyte (×10^9^/L)	1.26 ± 0.62		1.29 ± 0.79				0.625	
Lymphocyte (×10^9^/L)	0.50 ± 0.25		0.55 ± 0.29				0.099	
Neutrophil (%)	70.13 ± 11.54		70.49 ± 11.50				0.760	
Monocyte (%)	7.30 ± 2.37		7.74 ± 2.62				0.077	
Lymphocyte (%)	17.5(12.18–25.68)		16.25(12.58–25.58)				0.527	
TBIL (μmol/L)	8.70(6.30–12.10)		10.05(7.50–14.13)				***0*.*002***	
DBIL (μmol/L)	3.45(2.50–5.40)		3.55(2.38–5.60)				0.126	
IBIL (μmol/L)	4.80(3.40–7.03)		5.70(3.98–7.95)				***0*.*049***	
ALT (IU/L)	15.00(10.00–21.00)		28.00(15.75–38.00)				***<0*.*001***	
AST (IU/L)	19.50(16.00–25.00)		27.00(20.00–34.00)				***<0*.*001***	
TP (g/L)	68.82 ± 9.15		69.42 ± 8.42				0.508	
ALB (g/L)	37.89 ± 6.90		38.64 ± 7.35				0.248	
GLB (g/L)	30.93 ± 7.02		30.78 ± 6.65				0.829	
GLU (mmol/L)	5.14(4.71–5.89)		5.15(4.64–5.95)				0.41	
UREA (mmol/L)	4.05(3.15–5.30)		3.92(2.90–5.24)				0.299	
CREA (μmol/L)	60.45(49.00–73.20)		57.50(47.78–67.00)				0.601	
CYS-C (mg/L)	0.92(0.79–1.06)		0.91(0.81–1.04)				0.975	
Uric (μmol/L)	331.51 ± 155.30		291.29 ± 125.98				***0*.*008***	
TG (mmol/L)	1.06(0.80–1.43)		0.99(0.81–1.31)				0.469	
CHOL (mmol/L)	3.96 ± 1.058		3.96 ± 1.206				0.966	
HDL-C (mmol/L)	1.08(0.82–1.41)		1.12(0.85–1.48)				0.811	
LDL-C (mmol/L)	2.21(1.69–2.77)		2.20(1.79–2.72)				0.575	
ALP (IU/L)	79.00(64.00–98.00)		85.50(68.50–106.00)				***0*.*021***	
GGT (IU/L)	29.00(19.00–48.00)		42.50(26.00–78.00)				***<0*.*001***	
CRP (mg/L)	12.25(2.67–37.43)		9.74(2.30–39.23)				0.961	
ESR (mm/h)	33.50(14.75–64.00)		38.50(20.50–63.00)				0.173	
**Severity**			N	age	p	Gender (N)		p
					0.888	Male	female	0.117
mild			83	40.42±16.48		53	30	
moderate			21	42.19±14.04		11	10	
severe			14	41.57±14.78		5	9	

TB, tuberculosis; PTB, pulmonary tuberculosis; EPTB, extra-pulmonary tuberculosis.

### SNP alleles, genotypes, genetic models and haplotype analysis

The genotyping of selected SNPs was performed for all 118 patients in the ATDILI group and 628 patients in the non-ATDILI group. The concordance rate of the genotype results for the blinded repeated samples was 99.5%. None of the SNP genotype distributions deviated from Hardy-Weinberg equilibrium (HWE) (p>0.05 for all loci) (Table 2). The results for the rs3814055 SNP in PXR showed statistical significance; the proportion of the T allele was 37/199 (15.68%) in the ATDILI group and 291/959 (23.28%) in the non-ATDILI group when compared with the C allele (OR 0.61; 95% CI: 0.42–0.89, p = 0.01). The occurrence of the TT genotype was less common in the ATDILI group (3/118, 2.54%) compared with the non-ATDILI group (39/373, 6.24%), with a p value of 0.04. For both the rs78872571 and rs4647992 loci in NF-κB1, compared with the major allele (C), the minor allele (T) was significantly associated with increased ATDILI risk (OR 1.91; 95% CI: 1.06–3.43, p = 0.028 and OR 1.81;1.06–3.10, p = 0.029, respectively), as shown in Table 2. For rs13059232, rs7643645 and rs3732360 in PXR and rs60371688, rs1598861 and rs3774959 in NF-κB1, no allele or genotype differences were found between the two groups (all p>0.05). The distributions of the genotype and allele frequencies for all nine SNPs are depicted in Table 2.

**Table 2:** The distributions of genotype and allele frequencies for all nine SNPs.

Gene	dbSNP	N	allele	ATDILI (n,%)	non-ATDILI (n,%)	OR (95% CI)	P	P^HWE^	genotype	ATDILI	non-ATDIH	P
PXR	rs3814055	743	T	37(15.68)	291(23.28)	0.61(0.42–0.89)	<0.001	0.262	11	3	39	0.04		
	C>T		C	199(84.32)	959(76.72)	1			12	31	213			
									22	84	373			
	rs13059232	745	T	87(37.18)	479(38.14)	0.96(0.72–1.28)	0.78	0.612	11	18	88	0.65		
	T>C		C	147(62.82)	777(61.86)	1			12	51	303			
									22	48	237			
	rs7643645	744	G	99(42.30)	561(44.74)	0.91(0.68–1.20)	0.49	0.686	11	24	128	0.52		
	A>G		C	135(57.70)	693(55.26)	1			12	51	305		
									22	42	194		
	Rs3732360	746	G	99(41.95)	527(41.96)	0.99(0.75–1.82)	0.99	0.501	11	23	106	0.56	
	G>A		A	137(58.05)	729(58.04)	1			12	53	315		
									22	42	207		
NF-κB1	rs78872571	745	T	16(6.78)	46(3.67)	1.91(1.06–3.43)	**0.028**	0.366	11	1	1	0.07	
	C>T		C	220(93.22)	1208(96.33)	1			12	14	44		
									22	103	582		
	rs4647992	746	T	19(8.05)	58(4.62)	1.81(1.06–3.10)	**0.029**	0.439	11	2	1	**0.02**	
	C>T		C	217(91.95)	1198(95.38)	1			12	15	56		
									22	101	571		
	rs60371688	743	T	114(48.72)	606(48.40)	1.01(0.78–1.34)	0.929	0.557	11	30	140	0.49	
	T>C		C	120(51.28)	646(51.59)	1			12	54	326		
									22	33	160		
	rs1598861	743	C	28(11.97)	188(15.02)	0.77 (0.50–1.18)	0.224	0.660	11	3	14	0.29	
	A>C		A	206(88.03)	1064(84.98)	1			12	22	160		
									22	92	452		
	rs3774959	744	A	96(41.03)	544(44.18)	0.88 (0.66–1.17)	0.372	0.412	11	21	115	0.42	
	G>A		G	138(58.97)	700(55.82)	1			12	54	324		
									22	42	188		

P: p value was calculated using logistic regression analysis. PHWE: p value of the Hardy-Weinberg equilibrium. HWE was assessed by the χ2 goodness-of-fit test based on the genotype distributions in this study. Bold text means the p value<0.05.“1” designates the mutant allele and “2” designates the wildtype allele; 11 = mutant homozygote, 12 = heterozygote, 22 = wild homozygote

Three genetic models (dominant, recessive and additive pattern) were generated to determine the significance of each SNP. In line with the findings mentioned above, as shown in Table 3, rs3814055 (dominant model: OR 0.60; 95% CI: 0.39–0.92, p = 0.02; additive model: OR 0.62; 95% CI: 0.43–0.90, p = 0.01), rs78872571 (dominant model: OR 1.88; 95% CI: 1.01–3.51, p = 0.04; additive model: OR 1.88; 95% CI: 1.05–3.37, p = 0.03) and rs4647992 (dominant model: OR 1.68; 95% CI: 0.94–3.03, p = 0.08; additive model: OR 1.78; 95% CI: 1.05–3.03, p = 0.03) showed statistical significance. No genetic model was associated even marginally with ATDILI for the six other SNPs (rs13059232, rs7643645 and rs3732360 in PXR and rs60371688, rs1598861 and rs3774959 in NF-κB1).

**Table 3:** Genetic models of the association of the related SNPs with ATDILI in tuberculosis patients.

Gene	dbSNP	Dominant Model		Recessive Model		Additive Model	
PXR		OR (95% CI)	P value	OR (95% CI)	P value	OR (95% CI)	P value
	rs3814055	0.60 (0.39–0.92)	**0.02**	0.39 (0.12–1.29)	0.12	0.62(0.43–0.90)	**0.01**
	rs13059232	0.87(0.58–1.30)	0.50	1.12 (0.64–1.94)	0.70	0.96(0.72–1.28)	0.78
	rs7643645	0.80(0.53–1.21)	0.29	1.01 (0.62–1.64)	0.98	0.91(0.69–1.20)	0.50
	rs3732360	0.89(0.59–1.32)	0.49	1.19 (0.72–1.97)	0.49	1.00(0.75–1.33)	0.99
NF-)B1	rs78872571	1.88 (1.01–3.51)	**0.04**	5.35 (0.33–86.14)	0.27	1.88(1.05–3.37)	**0.03**
	rs4647992	1.68 (0.94–3.02)	0.08	10.81 (0.97–120.2)	0.05	1.78(1.05–3.03)	**0.03**
	rs60371688	0.87 (0.56–1.36)	0.55	1.97 (0.76–1.89)	0.44	1.01(0.76–1.34)	0.93
	rs1598861	0.77(0.44–1.14)	0.15	1.15 (0.33–4.07)	0.83	0.77(0.51–1.18)	0.23
	rs3774959	0.77(0.51–1.316)	0.20	0.97(0.58–1.63)	0.92	0.88(0.66–1.17)	0.36

P: p value was calculated using logistic regression analysis

We next generated the haplotypes to analyse whether there were additive associations among the selected SNPs with a pairwise r^2^>0.80. No haplotype could be generated for PXR. There was one haplotype for NF-κB1 consisting of rs60371688, rs1598861 and rs3774959 with a frequency = 0.055, which was associated with susceptibility to ATDILI (p = 0.01) (Table 4). The loci in the PXR and NF-κB1 genes within the linkage disequilibrium (LD) block are shown in S2 Fig.

**Table 4:** Analysis of haplotypes comprised of three polymorphisms in NF-κB1 and the risk of ATDILI.

rs60371688:rs1598861:rs3774959	Frequency	ATDILI, non-ATDILI Ratio*	P value
CAA	0.430	0.397,0.436	0.271
TAG	0.363	0.380,0.360	0.551
TCG	0.145	0.120,0.150	0.224
CAG	0.055	0.090,0.048	0.010

* Ratio is shown according to the CC frequencies

### Analysis of the association between SNPs and the severity of ATDILI

ATDILI cases were graded as mild (83/118, 70.33%), moderate (21/118, 17.80%), and severe (14/118, 11.86%) according to the WHO Toxicity Classification Standards. Age and gender were similar in the three groups (p = 0.888 and p = 0.117, respectively). We analysed the association between the three SNPs (rs7882571, rs4647992, rs3814055) and the risk of severity, and the results are shown in Table 5. Regrettably, no allele or genotype corresponding to the three SNPs was found to be associated with the severity of ATDILI. This may be due to the small sample sizes in the moderate and severe groups.

**Table 5:** Analysis of the association between the SNPs and ATDILI severity.

	Mild (N, %)			Moderate (N, %)			Severe (N, %)			P
	11	12	22	11	12	22	11	12	22	
rs7882571	1(1.20)	7(8.43)	75(90.3)	0(0)	3(14.2)	18(85.7)	0(0)	4(28.5)	10(71.4)	0.125
rs4647992	1(1.20)	9(10.8)	73(87.9)	0(0)	5(23.8)	16(76.1)	1(7.14)	1(7.14)	12(85.7)	0.517
rs3814055	1(1.20)	25(30.1)	57(68.6)	1(4.76)	3(14.2)	17(80.9)	1(7.14)	3(21.4)	10(71.4)	0.116
	1	2		1	2		1	2		P
rs7882571	9(0.05)	157(0.94)		3(0.07)	39(0.92)		4(0.14)	24(0.85)		0.146
rs4647992	11(0.06)	155(0.93)		5(0.11)	37(0.88)		3(0.10)	25(0.89)		0.235
rs3814055	27(0.16)	139(0.83)		5(0.11)	37(0.88)		5(0.17)	23(0.82)		0.794

“1” designates the mutant allele and “2” designates the wildtype allele

“11” designates mutant homozygote, “12” designates heterozygote, “22” designates wild homozygote

P: p value was calculated using Kruskal-Wallis one-way ANOVA (K independent samples)

### Joint effect analysis of the SNP-SNP interactions with ATDILI

The multiplicative interactions of each pair within the three positive SNPs were fitted using ordinal logistic regression to assess the within-pair independence, with two SNPs entering the logistic regression model at the same time. The additive interactions of the differential SNP loci (rs3814055 in PXR, rs78872571 and rs4647992 in NF-κB1) combined with the genotype distribution characteristics at positive sites were included in the analysis for the dominant or recessive model. None of the multiplicative interactions or the additive interactions reached the threshold value of statistical significance; all confidence intervals of the relative excess risk of interaction (RERI) and the attributable proportion of interaction (AP) contained 0, and the confidence interval of the synergy index (S) contained 1. The results for the determination of the additive interactions and multiplicative interactions between the positive SNPs are shown in Table 6.

**Table 6:** Analysis of the interactions between positive SNPs.

SNPs	Additive interactions				Multiplicative interactions	
	Measure	Estimate	Lower	Upper	P value	OR (95% CI)
rs78872571 and rs3814055	RERI	-7.907	-37.24	21.43	0.215	1.78(0.72–4.44)
	AP	-0.716	-3.013	1.581		
	S	0.560	0.135	2.230		
rs4647992 and rs3814055	RERI	-1.307	-11.808	9.194	0.592	0.76(0.28–2.08)
	AP	-0.248	-2.200	1.705		
	S	0.766	0.121	4.859		

RERI: Relative excess risk of interaction; AP: Attributable proportion of interaction; S: Synergy index

## Discussion

In the present study, we demonstrated the association between PXR and NF-κB1 polymorphisms and the risk of ATDILI. We revealed that the T allele of rs3814055 in PXR was significantly associated with a decreased risk for ATDILI, while the T alleles of rs78872571 and rs4647992 were significantly associated with an increased risk for ATDILI. However, there was no additive or multiplicative interaction between the positive SNPs.

We found that patients with the T allele (rs3814055) had an increased risk of ATDILI (OR 0.61; 95% CI: 0.42–0.89, P = 0.01), which was similar to the results of a study by Z. Zazuli et al[16]. However, the study by Z. Zazuli showed that patients with the TT genotype (rs3814055) had a greater risk of ATDILI (OR 8.89; 95% CI: 1.36–57.93, P<0.05). There are some possible reasons for this controversial finding, such as heterogeneous ethnic origins and different definitions of ATDILI. Additionally, as studies of association typically address moderate or small effects and hence require large sample sizes, a small bias arising from population stratification may be important[34], and the sample size was larger and there was no age or sex differences between patients and controls in our study. The change at rs3814055 from the C to T allele is functional. The TT genotype of rs3814055 has previously been shown to be associated with higher induction by rifampicin of CYP3A4 avtivity in a study by Zhang[35]. A change from a C to a T allele was associated with significantly greater transcriptional activity in a study by Manjul Rana[36], which indicates that the rs3814055 C/T polymorphism has a direct effect on the transcriptional upregulation of PXR. In another study of flucloxacillin-induced hepatotoxicity, the CC genotype (rs3814055) was associated with an increased risk of hepatocyte injury in the presence of the decreased expression of CYP3A4, which may result in a higher accumulation of unmetabolized toxic drugs and lead to hepatocellular injury [13]. Moreover, rifampicin-mediated PXR activation can function as a negative regulator of inflammation and immunity through the inhibition of the NF-κB signalling pathway[37]. As the NF-κB signalling pathway is activated in liver injuries and many diseases[19], we speculate that the upregulation of PXR may contribute to a potential protective mechanism against ATDILI by negatively regulating the NF-κB signalling pathway. These two hypothesized protective mechanisms are consistent with our results. In another study of the Chinese population, rs7643645 but not rs3814055 in PXR showed a statistically significant association[14]. This was somewhat different from our results. After reviewing the literature carefully, the definitions of ATDILI in the two studies are not completely identical; in the other study, ATDILI was defined by an ALT at least two-fold higher than the ULN and/or a combined increase in AST and total bilirubin at least two-fold higher than the ULN. Our research used a more stringent standard for ATDILI (as mentioned above), and our study subjects included only Han Chinese. All of the patients enrolled were Han Chinese, whose demographic characteristics are completely consistent with the SNP information obtained for the Beijing Han population, while the former study included some non-Han subjects. Some previous studies also revealed that female sex was a risk factor for liver injury. For example, two PXR SNPs (rs2461823 and rs7643645) and 2 haplotypes were found to influence the risk of ATDILI only in females[15]. However, no SNP in PXR was found to increase susceptibility to ATDILI in our female subgroup analysis of rs7643645 and rs13059232 (depicted in S2 Table). As rs2461823 and rs13059232 had a strong association with linkage disequilibrium in the haploid analysis (r^2^ = 1) (http://bioinfo.bjmu.edu.cn/mirsnp/search/), we suspected that rs2461823 was not associated with susceptibility to ATDILI in females. Perhaps the discrepancy was due to differences in population composition; in our study, the population in the ATDILI and non-ATDILI groups did not show a gender difference (p = 0.285), while in the former study, the population was marginally characterized by a gender difference (p = 0.051)[15].

In our study, the T allele at the rs78872571 and rs4647992 loci was significantly associated with an increased risk for ATDILI. No study on rs78872571 has been reported yet. Although rs4647992 has been researched in studies of the pathway-targeted pharmacogenomics of the cytochrome enzyme P4501A2 in human liver and the common variations in genes related to innate immunity and the risk of adult glioma[38, 39], no correlation was found. We also examined the sites that potentially have a strong association with linkage disequilibrium by using haploid analysis (r^2^ = 1) (http://bioinfo.bjmu.edu.cn/mirsnp/search/); none of the related loci have exact functional overlap. Since these two loci are both located in the intron region, it is uncertain whether they can affect the transcription of NF-κB1 itself and alter the activity of p50 by alternative splicing. As is well known, reactive oxygen species, free radicals and various inflammatory factors produced during ATDILI development can directly activate NF-κB and cause further upregulation of the expression of NF-κB signalling pathway-related target genes, such as TNF-a, interleukin-2 (IL-2), interferon (INF) and chemokines. These inflammatory factors further mediate inflammatory damage in liver cells through cascade reactions[40, 41]. As an important molecule involved in the NF-κB signalling pathway, p50 has anti-inflammatory properties as part of the p50/p50 homodimer by repressing transcription, since it lacks the COOH-terminal trans-activation domain that is necessary for the regulation of gene expression. Meanwhile, as part of the p65/p50 complex, it has pro-inflammatory properties by controlling the transcription of pro-inflammatory cytokines. The relative abundances of the p65/p50 heterodimers and p50 homodimers may determine the degree of inflammation by balancing pro-inflammatory and anti-inflammatory responses[42]. This means that the transcription of NF-κB1 exerts two-way regulation of inflammatory responses. Can the variants further regulate the inflammatory response by disturbing P50 transcription?

Given that the combined analyses of SNPs may display a more complete picture of the candidate genes, we conducted a haplotype analysis and SNP-SNP interaction analysis of the selected positive SNPs. We found that only one haplotype in NF-κB1 (CAG) increased the risk of ATDILI without any multiplicative or additive interaction. Nevertheless, there is evidence that the interaction between the nuclear factor kappa B (NF-κB)-mediated inflammatory pathway and the pregnane X receptor (PXR)-regulated detoxification pathway maintains the homeostatic state of the intestine[43]. Endoplasmic reticulum stress can inhibit PXR transcription while activating NF-κB transcription, although the mechanism of interaction is unclear[44]. Rs3814055 is present in the 1139 bp region upstream of the transcription start site and may be related to the capability for transcription factor binding in the promoter region. Through bioinformatics analysis, 7 putative binding sites for NF-κB1 were predicted in the promoter sequence of PXR at a setting of 80% (http://jaspardev.genereg.net/). By combining our research results with a literature review, we speculated NF-κB1 may play a role in ATDILI by regulating PXR transcription, and this needs further functional verification. To the best of our knowledge, this is the first report on an association between variation in NF-κB1 and ATDILI. Furthermore, we analysed the association between the three SNPs (rs7882571, rs4647992, rs3814055) and the severity of ATDILI. No allele or genotype of the three SNPs was found to be associated with the severity of ATDILI. This may be due to the small sample sizes in the moderate and severe groups.

There are several strengths of our study. First, our prospective study included patients from West China Hospital, the largest medical centre in western China that conducts surveillance of ATDILI with strict criteria to avoid misclassification. Our study also had an acceptable sample size and stricter inclusion and exclusion criterion. We excluded people with hepatitis B virus or hepatitis C virus as well as HIV co-infection, which have been shown to be risk factors for ATDILI. Second, the researchers who collected and sorted the clinical data and those who were responsible for the laboratory analysis worked independently in this study to minimize potential bias. These differences may make the conclusions of the study more persuasive and representative to some degree.

There were several limitations in our study. First, we assessed only ATDILI induced by first-line anti-tuberculosis regimens without the evaluation of the second-line drugs. Second, we focused on only the genetic risk factors PXR and NF-κB1; the examination of other relevant genes, environmental risks, comorbid conditions (malnutrition, alcoholism, chronic hepatitis C and chronic hepatitis B infection, HIV infection and pre-existing liver disease) and epigenetic modifications is necessary to improve the accuracy and reliability of the study. Furthermore, extended validation in multicenter studies with enlarged samples in other cohorts is needed to identify the association between the targets and ATDILI and performed functional verification tests in vitro and vivo.

In conclusion, we found genetic polymorphisms in rs3814055 in PXR and rs78872571 and rs4647992 in NF-κB1 that were associated with susceptibility to ATDILI resulting from first-line anti-tuberculosis combination treatment in a Chinese population. We believe that mapping the PXR and NF-κB1 variants in a larger population, along with functional verification, will further explore the important role of PXR and the NF-κB signalling path in the development of ATDILI.

## Supporting information

S1 Fig Flow diagram of the study population. The process of enrolment in our study containing the inclusion and exclusion of all the case.
(DOCX)

S2 Fig Linkage disequilibrium (LD) plot of SNPs. The loci in the PXR and NF-κB1 genes within the linkage disequilibrium (LD) block
(DOCX)

S1 Table Candidate single nucleotide polymorphism of PXR and NF-κB1. Information of the genotyped SNPs containing the chromosomal locations and minor allele frequencies
(DOCX)

S2 Table Association between related SNPs with risk of ATDILI in female patients. The distributions of genotype and allele frequencies in female subgroup.
(DOCX)
