# Supplementary material for: Genetic polymorphisms in PXR and NF-κB1 influence susceptibility to anti-tuberculosis drug-induced liver injury
Source: PLoS One. 2019 Sep 6;14(9):e0222033. doi: 10.1371/journal.pone.0222033 (PMC6730870; doi:10.1371/journal.pone.0222033)
Supplement: S1 Fig — The process of enrolment in our study containing the inclusion and exclusion of all the case. (DOCX) [file pone.0222033.s001.docx]

**S1 Fig. Flow diagram of the study population.**

Highly suspicious TB patients were consecutively enrolled from December 2016 to April 2018(n=1060)

Excluded (n=287 )

  without evidenced of tuberculosis (n= 243 )

  with HIV,HBV or HCV (n=16 )

  with abnormal liver function before treatment (n=28 )

## Enrollment

Tuberculosis patients without anti-tuberculosis induced liver injury (n=628 )

Tuberculosis patients with anti-tuberculosis induced liver injury (n=118 )

## Analysis

Included with definite diagosis of tuberculosis

Six months follow-up treatment and data recording (n= 773 )

Excluded (n=27 )

  lost follow up (n= 13 )

  with use of other hepatotoxic medicine (n=16 )

  use second-line anti-tuberculosis drugs (n=28 )

Receive standard anti-tuberculosis drugs

With good treatment Compliance

(n=746 )
