# Supplementary material for: Genetic polymorphisms in PXR and NF-κB1 influence susceptibility to anti-tuberculosis drug-induced liver injury
Source: PLoS One. 2019 Sep 6;14(9):e0222033. doi: 10.1371/journal.pone.0222033 (PMC6730870; doi:10.1371/journal.pone.0222033)
Supplement: S2 Fig — The loci in the PXR and NF-κB1 genes within the linkage disequilibrium (LD) block (DOCX) [file pone.0222033.s002.docx]

**S2 Fig. Linkage disequilibrium (LD) plot of SNPs.**

| 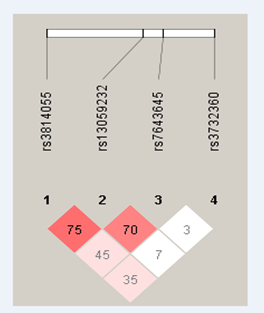 | 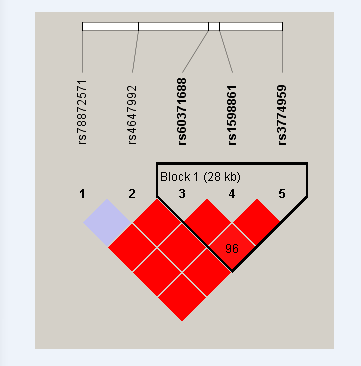 |
| --- | --- |
| A Linkage disequilibrium (LD) plot of 4 SNPs of PXR. | B Linkage disequilibrium (LD) plot of 5 SNPs of NF-κB1 |

Strong LD is represented by the high linkage disequilibrium correlation coefficient.
