# Supplementary material for: Genetic polymorphisms in PXR and NF-κB1 influence susceptibility to anti-tuberculosis drug-induced liver injury
Source: PLoS One. 2019 Sep 6;14(9):e0222033. doi: 10.1371/journal.pone.0222033 (PMC6730870; doi:10.1371/journal.pone.0222033)
Supplement: S1 Table — Information of the genotyped SNPs containing the chromosomal locations and minor allele frequencies (DOCX) [file pone.0222033.s003.docx]

**S1 Table. Candidate single nucleotide polymorphism of PXR and NF-κB1.**

| gene | dbSNP | allele | Location (GRCh38.p7) | region | MAF | MAF* |
| --- | --- | --- | --- | --- | --- | --- |
| PXR | rs3814055 | C>T | chr3:119781188 | 5 PrimeUTR Variant | 0.322 | 0.221 |
| PXR | rs13059232 | C>T | chr3:119802208 | Intron Variant | 0.359 | 0.380 |
| PXR | rs7643645 | A>G | chr3:119806650 | Intron Variant | 0.431 | 0.444 |
| PXR | rs3732360 | A>G | chr3:119817734 | 3 Prime UTR Variant | 0.411 | 0.420 |
| NF-κB1 | rs78872571 | C>T | Chr4:102512308 | Intron Variant | 0.034 | 0.042 |
| NF-κB1 | rs4647992 | C>T | Chr4:102534190 | Intron Variant | 0.043 | 0.052 |
| NF-κB1 | rs60371688 | T>C | Chr4:102561296 | Intron Variant | 0.481 | 0.485 |
| NF-κB1 | rs1598861 | A>C | Chr4:102565540 | Intron Variant | 0.116 | 0.145 |
| NF-κB1 | rs3774959 | G>A | Chr4:102589957 | Intron Variant | 0.454 | 0.437 |

MAF: minor allele frequency in 1000 Genomes (East Asia) <https://www.ncbi.nlm.nih.gov/snp>.

*: MAF Calculated by Haploview software in our study
