# Supplementary material for: Genetic polymorphisms in PXR and NF-κB1 influence susceptibility to anti-tuberculosis drug-induced liver injury
Source: PLoS One. 2019 Sep 6;14(9):e0222033. doi: 10.1371/journal.pone.0222033 (PMC6730870; doi:10.1371/journal.pone.0222033)
Supplement: S2 Table — The distributions of genotype and allele frequencies in female subgroup. (DOCX) [file pone.0222033.s004.docx]

**S2 Table. Association between related SNPs with risk of ATDILI in female patients.**

|  |  | Genotype | P | Allele | | | additive model | | | Dominant model | | | Recessive model | | |
| --- | --- | --- | --- | --- | --- | --- | --- | --- | --- | --- | --- | --- | --- | --- | --- |
| SNP | group | (11/12/22） | | OR | 95%CI | P | OR | 95%CI | P | OR | 95%CI | P | OR | 95%CI | P |
| rs3814055 | ATDILI | 2/10/37 | 0.15 | 0.56 | (0.31- 1.03) | 0.06 | 0.58 | (0.32-1.05) | 0.07 | 0.50 | (0.25-1.01) | 0.05 | 0.63 | (0.14-2.82) | 0.54 |
|  | Non-ATDILI | 16/83/153 |  |  | |  |  | | |  | | |  | | |
| rs13059232 | ATDILI | 8/18/23 | 0.25 | 0.82 | (0.52-1.29) | 0.39 | 0.82 | (0.52-1.29) | 0.39 | 0.64 | (0.34-1.18) | 0.15 | 1.14 | (0.49-2.62) | 0.76 |
|  | Non-ATDILI | 37/125/91 |  |  | |  |  | | |  | | |  | | |
| rs7643645 | ATDILI | 10/21/18 | 0.72 | 0.87 | (0.56-1.35) | 0.54 | 0.88 | (0.57-1.34) | 0.55 | 0.77 | (0.41-1.46) | 0.43 | 0.94 | (0.44-2.01) | 0.87 |
|  | Non-ATDILI | 54/120/78 |  |  | |  |  | | |  | | |  | | |
| rs3732360 | ATDILI | 13/17/19 | 0.47 | 1.18 | (0.76-1.82) | 0.47 | 1.16 | (0.76-1.78) | 0.48 | 0.92 | (0.49-1.72) | 0.79 | 1.81 | (0.89-3.71) | 0.10 |
|  | Non-ATDILI | 42/118/93 |  |  |  |  |  |  |  |  |  |  |  |  |  |

“1” designates the mutant allele and “2” designates the wild allele;“11” designates mutant homozygote, “12” designates heterozygote, “22” designates wild homozygote;
